# Supplementary figures and images for: Structural analysis of the dynamic ribosome-translocon complex
Source: eLife. 2024 Jun 18;13:RP95814. doi: 10.7554/eLife.95814 (PMC11186639; doi:10.7554/eLife.95814)

Figure 3F -- source data 2

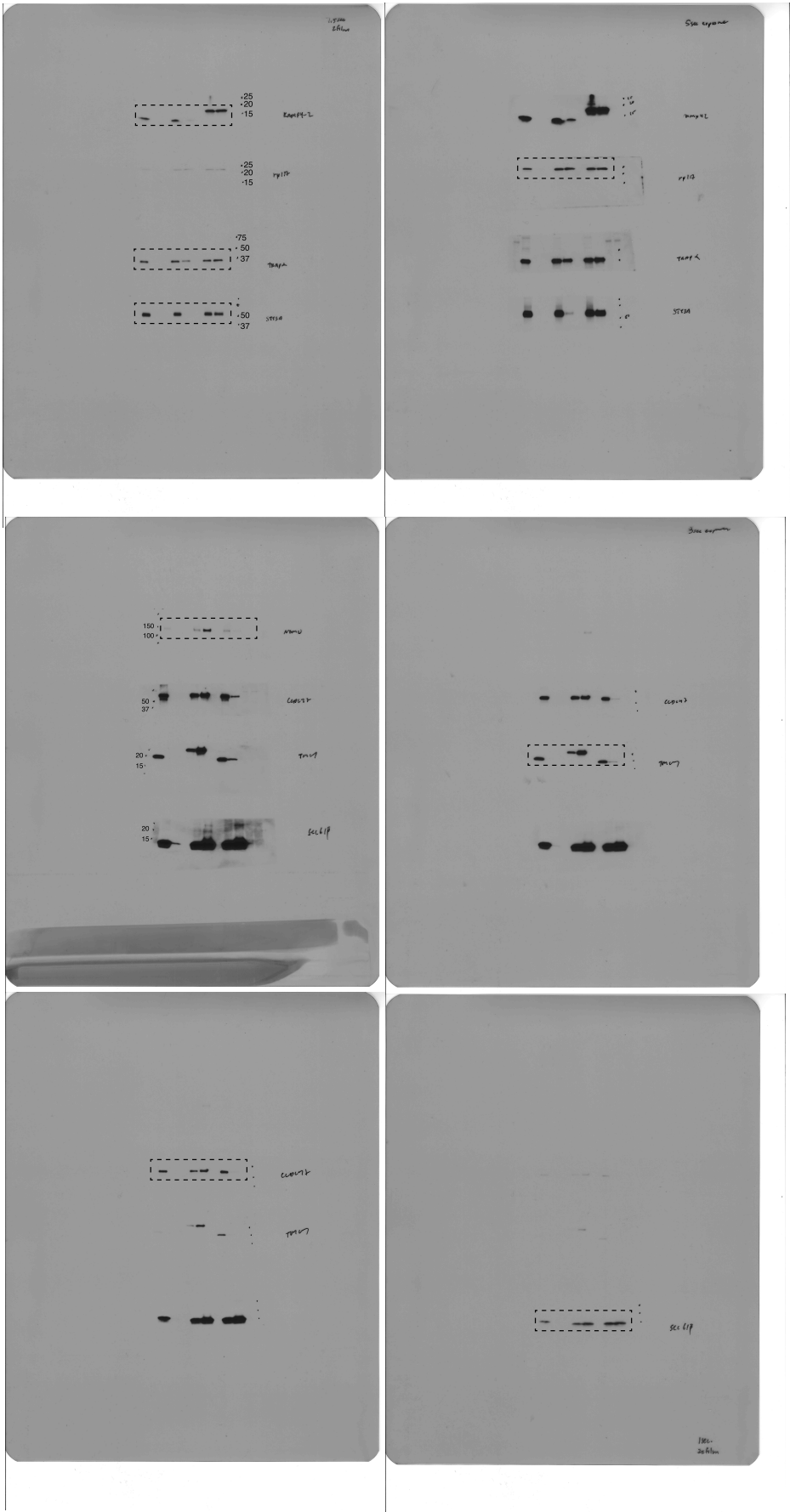

Supplement: Figure 3—source data 1. [file elife-95814-fig3-data1.zip › Figure 3-source data 1/Fig 3F source data 2.pdf]

Figure 3F -- source data 1

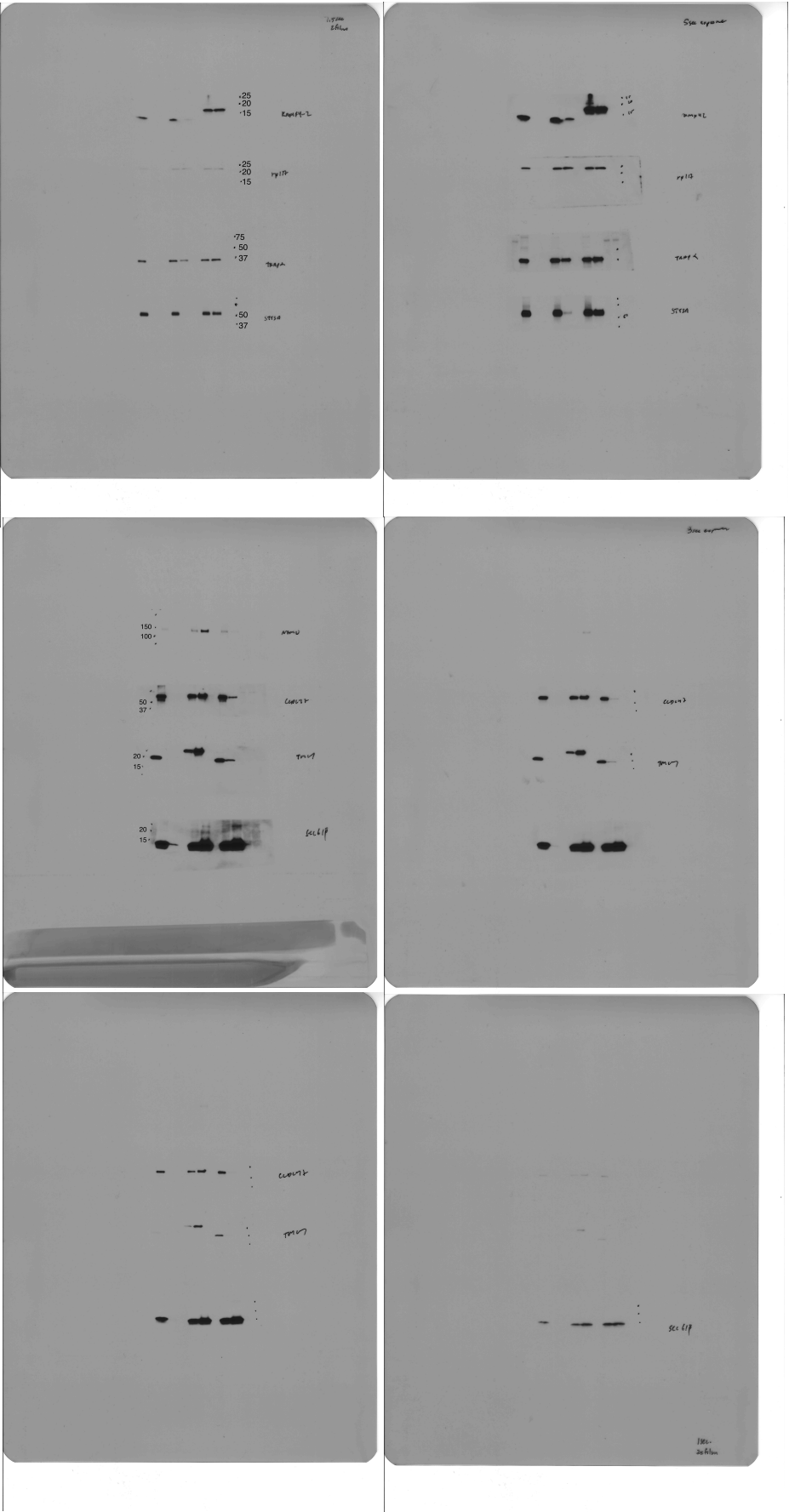

Supplement: Figure 3—source data 1. [file elife-95814-fig3-data1.zip › Figure 3-source data 1/Fig 3F source data 1.pdf]
